# Relapse of acute malnutrition and associated factors after discharge from nutrition stabilization centers among children in Eastern Ethiopia

**DOI:** 10.3389/fnut.2023.1095523

**Published:** 2023-02-14

**Authors:** Mohammedjemal Alyi, Kedir Teji Roba, Indeshaw Ketema, Sisay Habte, Abel Tibebu Goshu, Ame Mehadi, Yohannes Baye, Behailu Hawulte Ayele

**Affiliations:** ^1^Habro Woreda Health Office, Gelemso, Ethiopia; ^2^School of Nursing and Midwifery, College of Health and Medical Sciences, Haramaya University, Harar, Ethiopia; ^3^School of Public Health, College of Health and Medical Sciences, Haramaya University, Harar, Ethiopia

**Keywords:** relapse, acute malnutrition, stabilization center, children, Habro Woreda, Eastern Ethiopia

## Abstract

**Background:**

Acute malnutrition is a major global health problem primarily affecting under-five children. In sub-Saharan Africa, children treated for severe acute malnutrition (SAM) at an inpatient have high case fatality rate and is associated with relapse of acute malnutrition after discharge from inpatient treatment programs. However, there is limited data on the rate of relapse of acute malnutrition in children after discharge from stabilization centers in Ethiopia. Hence, this study aimed to assess the magnitude and predictors of relapse of acute malnutrition among children aged 6–59 months discharged from stabilization centers in Habro Woreda, Eastern Ethiopia.

**Methods:**

A cross-sectional study was conducted among under-five children to determine the rate and predictors of relapse of acute malnutrition. A simple random sampling method was used to select participants. All randomly selected children aged 6–59 months discharged from stabilization centers between June 2019 and May 2020 were included. Data were collected using pretested semi-structured questionnaires and standard anthropometric measurements. The anthropometric measurements were used to determine relapse of acute malnutrition. Binary logistic regression analysis was used to identify factors associated with relapse of acute malnutrition. An odds ratio with 95% CI was used to estimate the strength of the association and a *p*-value less than 0.05 was considered statistically significant.

**Results:**

A total of 213 children with mothers/caregivers were included in the study. The mean age in months of children was 33.9 ± 11.4. More than half (50.7%) of the children were male. The mean duration of children after discharge was 10.9 (± 3.0 SD) months. The magnitude of relapse of acute malnutrition after discharge from stabilization centers was 36.2% (95% CI: 29.6,42.6). Several determinant factors were identified for relapse of acute malnutrition. Mid-upper arm circumference less than 110 mm at admission (AOR = 2.80; 95% CI: 1.05,7.92), absence of latrine (AOR = 2.50, 95% CI: 1.09,5.65), absence of follow-up visits after discharge (AOR = 2.81, 95% CI: 1.15,7.22), not received vitamin A supplementation in the past 6 months (AOR = 3.40, 95% CI: 1.40,8.09), household food insecurity (AOR = 4.51, 95% CI: 1.40,15.06), poor dietary diversity (AOR = 3.10, 95% CI: 1.31,7.33), and poor wealth index (AOR = 3.90, 95% CI: 1.23,12.43) were significant predictors of relapse of acute malnutrition.

**Conclusion:**

The study revealed very high magnitude of relapse of acute malnutrition after discharge from nutrition stabilization centers. One in three children developed relapse after discharge in Habro Woreda. Programmers working on nutrition should design interventions that focus on improving household food insecurity through strengthened public Safety Net programs and emphasis should be given to nutrition counseling and education, as well as to continuous follow-up and periodic monitoring, especially during the first 6 months of discharge, to reduce relapse of acute malnutrition.

## Introduction

Malnutrition is a major public health challenge faced by many developing countries (1, 2). It occurs when food intake is not balanced with the body’s dietary needs, particularly in vulnerable groups such as under-five children, pregnant and lactating mothers, and people with chronic illness (3, 4). It is a general term representing both undernutrition and overnutrition (5). Undernutrition contributes significantly to the global disease burden and is responsible for 35% of deaths in children (1).

Acute malnutrition is a life-threatening condition generally resulting from the recent rapid loss of weight or a failure to gain weight due to illness, reduced food intake, and inappropriate childcare practices or combinations of these factors (6). It is determined by the nutritional index of weight-for-height (WFH) and/or mid-upper arm circumference (MUAC) and categorized into moderate acute malnutrition (MAM) and severe acute malnutrition (SAM) (7). SAM is one of the top nutrition-related causes of death in children worldwide (8). Globally, over 49 million under-five children were wasted, and nearly 17 million were severely wasted, with the largest number being found in South Asia (68%) and Africa (28%) (9, 10).

In Ethiopia, SAM is a serious public health problem that poses a significant obstacle to achieving better child health outcomes (11). Despite a substantial decline in the proportion of wasting (7%), stunting (37%), and underweight (21%) in the last two decades, undernutrition in children is still a common problem in Ethiopia (12–14). A previous study conducted in Haramaya district, Ethiopia indicated that about 14, 36, and 23% of children were wasted, stunted and underweighted, respectively (15).

According to the community-based management of acute malnutrition (CMAM) guidelines, children with failed appetite, and/or with a major medical complication and infants under 6 months with bilateral pitting edema or visible wasting should be initially admitted to inpatient treatment programs. Whereas those children with a good appetite, no major medical complications or edema, and no marasmic-kwashiorkor should be linked to outpatient treatment programs (OTP) (16). The protocol for the management of acute malnutrition also recommends that children should be periodically monitored after discharge from the treatment programs (1, 2).

The literature shows that high mortality rate of SAM in children after being discharged from inpatient treatment programs (17–20). Several factors are identified for the relapse, including living in settings of pervasive poverty, low women literacy, food insecurity, inadequate water and sanitation, incomplete vaccination and poor follow-up after discharge (21–23).

In sub-Saharan Africa, children treated as an inpatient with SAM have 10 to 40% case fatality (24). A previous study in Ethiopia showed that 29% of children treated in a Stabilizing center died from SAM (25). Evidence indicates that SAM children discharged as recovered may relapse following exit from CMAM programs (26). For instance, a previous study conducted in Ethiopia showed that 34% of children relapse after being discharged from a CMAM program (27).

Despite the high burden of acute malnutrition in Ethiopia, there is limited data on the rate of relapse of acute malnutrition among children discharged from healthcare facilities after being managed for nutritional problems. Therefore, this study aimed to determine the prevalence of relapse of acute malnutrition and associated factors among 6–59 months old children after discharge from stabilization centers in Habro Woreda, Eastern Ethiopia.

## Materials and methods

### Study setting and period

The study was conducted in Habro Woreda from December 15, 2020 to January 15, 2021. Habro Woreda is one of the 15 woredas in the West Hararghe zone of Oromia Regional State, Eastern Ethiopia. Its administrative office is found in Gelemso town, which is located 400 km from Addis Ababa, the capital city of Ethiopia, to the east. The Woreda has 37 kebeles and an estimated total population of 269,279, with an estimated 44,242 under-five children and 55,100 households. There are 7 health centers and 1 general hospital providing nutrition stabilization services in the woreda.

### Study design and population

A cross-sectional study was employed to determine the rate of relapse of acute malnutrition and associated factors among children aged 6–59 months previously treated for SAM and discharged as recovered between June 2019 and May 2020 in Habro Woreda. The source population were children aged 6–59 months discharged recovered from SC in Habro Woreda and the study population were those 6–59 months old children following discharge as recovered in the randomly selected kebeles of Habro Woreda. All randomly selected children aged 6–59 months who were discharged recovered 6 months prior to the data collection period with mothers/caregivers who gave consent were included. Children who had incomplete address information on the SC registration logbooks and children with physical deformities during anthropometric measurements were excluded.

### Sample size determination

The sample size for the first objective was determined using a single population proportion formula with the assumptions of a 15.4% prevalence of relapse of acute malnutrition from a previous study (23), 95% CI, 5% margin of error and 10% non-response rate, yielding the sample size of 220. The sample size for the second objective was calculated using Epi–info version 7.0 by considering various factors associated with relapse of acute malnutrition including the educational status of women (28), follow-up visits after discharge (29), vitamin A supplementation within the last 6 months (27) and pre-lacteal feeding (27) with the assumptions of 95% confidence level, power of 80% and 10% non-response rate, yielding the sample size of 130, 143, 224, and 156, respectively. Finally, the final sample size required for this study was determined by taking the largest sample size observed in the second objective which was 224.

### Sampling techniques and procedures

Out of 37 kebeles found in Habro Woreda, 20 kebeles were selected randomly (by lottery method). All malnutrition cases previously admitted for SAM treatment and discharged from seven health centers (Balbeletti, Firi Jiru, Dereku, Gelemso, Wayne Gudo, Ceffe, and Wachu) and Gelemso General Hospital in the selected kebeles of Habro Woreda were traced from SAM records/registration logbooks. The total records of SAM children discharged from SC in selected kebeles with complete information were 297. The total cases of all records of SAM children were allocated to each selected kebele based on their addresses from registration logbooks. The households of each study participant were accessed with health extension workers (HEW) working in each kebele using lists of addresses from registration logbooks. The sampling frame was prepared using children’s serial numbers obtained from registration logbooks. Finally, after proportionally allocating the sample size to each selected kebele based on the number of malnutrition cases, the study participants were selected using a simple random sampling technique.

### Data collection tools and techniques

A pretested validated semi-structured questionnaire adapted from relevant literature (27, 30, 31) and tailored to the study variables was used to collect data. The questionnaire was initially developed in English and translated to the local languages (Afan Oromo and Amharic) and then re-translated into English by language experts to check for consistency. The questionnaire has six (6) parts: Socioeconomic and demographic characteristics (age, sex, place of residence, family size, mother’s occupation, educational status of parents, and family wealth index), anthropometric measurements, previous clinical characteristics, child feeding characteristics, household food security status and housing conditions. Data were collected by trained data collectors and supervisors through face-to-face interviews with mothers/caretakers and anthropometric measurements. Information on clinical data during the treatment period was collected retrospectively from SAM records. To identify the history of morbidity, mothers were asked for any occurrence of illness during the past 2 weeks. Children’s vaccination status was checked by observing their immunization card and if not available, mothers were asked to recall the child’s vaccination. BCG vaccination was also checked if any scar existed on a child’s arm.

### Measurement of variables

#### Relapse

Was considered in a child whose MUAC is <125 mm and/or WFH < −2SD and/or the presence of bilateral pitting edema after discharge from inpatient treatment programs. Children whose WFH is < −2SD and/or MUAC 115 to <125 mm were categorized as MAM, whereas children whose WFH < −3SD and/or MUAC < 115 mm were considered SAM (32).

#### Anthropometric measurements

Height and weight were measured following standard techniques by using calibrated instruments, and recorded to the nearest 0.1 cm and 0.1 kg, respectively. The height for children less than 2 years was measured by gently placing a child on his/her back in the middle of the board, facing straight up with shoulder blades, buttocks, and heels touching the surface of the height board and knees fully straight, arms stretched on the child’s sides and feet at right angles. Whereas for children 2 years or older, was measured while a child is standing upright in the middle of the height board with arms at the sides, knees straight and the child’s head, shoulders, buttocks and heels touching the board, and feet close together (16, 33–35). MUAC was measured following recommended steps using a color-coded tape. Presence of edema was assessed, recorded and categorized using recommended procedures. Children whose WFH is below minus two standard deviations (−2SD) and/or MUAC < 125 mm, with/without pitting edema were considered acute malnutrition (7, 32, 36).

#### Dietary diversity score (DDS)

Was determined by summing different food groups consumed using a 24-h recall period. A total of eight food groups were used to guide the scoring as per food items consumed. The participants received “1” point if they consumed a minimum of one food item within each subgroup, and “0” point if they did not. Finally, the DDS was computed and categorized as high for a score ≥6, medium for a score of 4–5, and low for a score of ≤3 (37).

#### Household food security status

Was determined using nine standard Household Food Insecurity Access Scale (HFIAS) questions: an occurrence question followed by the frequency of occurrence of the events. Prior to assigning the food insecurity access, each frequency of occurrence responses was coded as “0” for all cases where the answer to the corresponding occurrence question was “no” and then the four food security categories were created sequentially as recommended by Food and Nutrition Technical Assistance (FANTA) (38). Finally, the HFIAS category one (1) was considered as food secure and the remaining as food insecure.

#### Family wealth index

Was computed using Principal Component Analysis (PCA) as a composite indicator of household living standards. Finally, the wealth tercile was performed and categorized as higher, medium, and lower (39, 40).

### Data quality management

A pretested validated semi-structured data collection tool was adopted after a review of related literature to ensure data quality. Two days training were given to data collectors and supervisors on the objectives of the study, the contents of data collection tools, anthropometric measurements, and how to collect and record data appropriately. A pretest was conducted on 5% of the sample size before the actual data collection period to check for the reliability and validity of data collection tools. The collected data were carefully checked for completeness, accuracy and consistency by supervisors and the principal investigator on daily basis. Double data entry was done by two individuals to minimize errors.

### Data processing and analysis

The collected data were categorized, coded and entered into Epi-Data version 3.1 and analyzed using STATA software version 14.2. Texts, tables, and figures were used to display descriptive and summary statistics. Binary logistic regression analysis was conducted to identify factors associated with relapse of acute malnutrition. Initially, bivariate logistic regression analysis was conducted to determine the candidate variables for the multivariate logistic regression analysis. The Logistic regression model fitness was checked using Hosmer-Lemeshow and indicates fitted for the model. Multicollinearity was checked (VIF < 10) indicating non-existence of multicollinearity among variables. Variables with a *p*-value < 0.20 were fitted into a multivariate logistic regression analysis to identify factors significantly associated with relapse of acute malnutrition. Both crude and adjusted odds ratio along with 95% CI was estimated to measure the strength of the association. Finally, a *p*-value < 0.05 at 95% confidence interval (CI) was considered statistically significant.

## Results

### Socioeconomic and demographic characteristics of study participants

**FIGURE 1:**
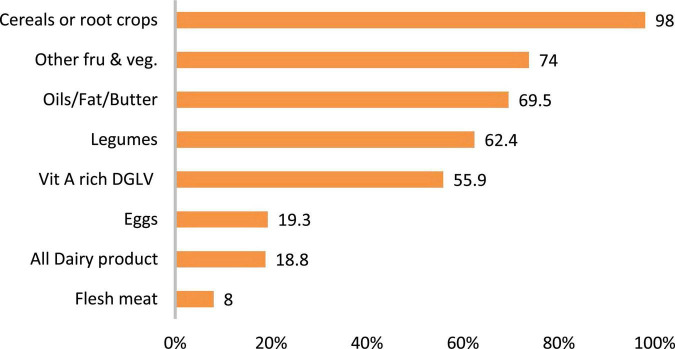
Percentage of food groups consumed by the children in the past 24-h in Habro Woreda, Eastern Ethiopia, 2021 (*n* = 213). DGLV, dark green leafy vegetables.

A total of 213 children aged 6–59 months with mothers/caregivers were included in the study, giving a response rate of 95.1%. The majority (34.3%) of children were aged 36–47 months with a mean age of 33.9 ± 11.4 months. More than half (50.7%) of the children were male and the majority (83.1%) were rural residents. Regarding mother related variables, more than a quarter (30.9%) were in the age group of 30–34 years with a mean age of 31.3 ± 5.3 years. Nearly three-fourths (74.6%) of mothers were homemakers and the majority (86.8%) had never attended formal education. In this study, 28.6 and 10.8% of the households were mildly and severely food insecure, respectively. The mean dietary diversity score of participants was 4.1 (± 1.3 SD). In the current study, 46% of children ate less than four food groups in the preceding 24-h (Table 1). The most commonly consumed food groups were cereals (98%), fruits and vegetables (74%), legumes (62.4%), and dark green leafy vegetables (55.9%) (Figure 1).

**TABLE 1:** Socioeconomic and demographic characteristics of the study participants in Habro Woreda, Eastern Ethiopia, 2021 (*N* = 213).

Variables	Category	Frequency	Percent
Age of child (in months)	12–23	45	21.1
	24–35	65	30.5
	36–47	73	34.3
	48–59	30	14.1
Residence of a child	Urban	36	16.9
	Rural	177	83.1
Sex of child	Male	108	50.7
	Female	105	49.3
Age of mother (in years)	20–24	24	11.3
	25–29	53	24.9
	30–34	66	30.9
	35–39	59	27.7
	40–44	11	5.2
Sex of the household head	Male	190	89.2
	Female	23	10.8
Marital status of a mother	Married	190	89.2
	Unmarried	23	10.8
Occupation of mothers	Homemaker	159	74.6
	Daily labor	27	12.7
	Farmer	18	8.5
	Merchant	9	4.2
Maternal educational status	No formal education, unable to read and write	146	68.5
	No formal education, can read and write	39	18.3
	Primary education (Grade 1–8)	25	11.7
	Secondary education (Grade 9–12)	3	1.5
Paternal educational status (*n* = 190)	No formal education, unable to read and write	105	55.3
	No formal education, can read and write	52	27.4
	Primary (Grade 1–8)	20	10.5
	Secondary (Grade 9–12)	13	6.8
Total family size in the HH	1–3 members	16	7.5
	4–6 members	108	50.7
	≥7 members	89	41.8
Number of under-five children in the HH	One	64	30.1
	Two	131	61.5
	Three and above	18	8.4
Family wealth index	Poor	71	33.3
	Medium	71	33.3
	Rich	71	33.3
Household food security status	Food secure	45	21.1
	Mildly insecure	61	28.6
	Moderately insecure	84	39.4
	Severely insecure	23	10.8
Dietary diversity score	High DDS (>5)	22	10.3
	Medium DDS (4–5)	93	43.7
	Low DDS (<4)	98	46.0

DDS, dietary diversity score; HH, household.

### Clinical characteristics of children

The mean duration of study participants after discharge was 10.9 ± 3.0 months. Among the study participants, more than a quarter (27.7%), 47.9 and 11.3% of children had MUAC < 110 mm, severe marasmic and bilateral pitting edema at admission, respectively. The length of stay in the facility was ranging from 3–22 days with an average of 8.4 ± 3.3 days. In this study, nearly half (48.4%) of the children were discharged in the first week of admission. Of discharged children, the majority (88.3%) were transferred to the OTP and only 42.3% of the children were visited by healthcare workers after discharge. Regarding the immunization status, 74.7% of children were fully vaccinated, and 67.1% received vitamin A supplementation in the preceding 6 months (Table 2).

**TABLE 2:** Clinical characteristics of children discharged from SAM stabilization centers in Habro Woreda, Eastern Ethiopia, 2021 (*N* = 213).

Variables	Category	Frequency	Percent
Time beyond SC discharge (in months)	6–9	70	32.8
	10–12	92	43.2
	13–15	33	15.5
	16–18	18	8.5
Health facility to which a child is admitted	Gelemso hospital	35	16.43
	Gelemso HC	35	16.43
	Firi Jiru HC	32	15.02
	Wechu HC	32	15.02
	Chefe HC	25	11.74
	Dereku HC	38	17.84
	Balbeletti HC	9	4.23
	Wayne Gudo HC	7	3.29
Type of SAM at admission	Marasmic	102	47.9
	Kwashiorkor	89	41.8
	Marasmic-kwashiorkor	22	10.3
MUAC at admision	<110 mm	59	27.7
	110–115 mm	61	28.6
	>115 mm	93	43.7
Presence of edema at admission	Yes	24	11.3
	No	189	88.3
Weight gain during discharge from SC program	<5 g/kg/day	57	26.8
	5–10 g/kg/day	112	52.6
	>10 g/kg/day	44	20.6
Length of stay in the SC program (in days)	<7	103	48.4
	7–14	99	46.5
	>14	11	5.1
The child was transferred to which program after discharge	OTP	188	88.3
	OTP and SFP	21	9.8
	SFP	4	1.9
Adherence to the prescribed schedule	Yes	190	89.2
	No	23	10.8
Visited by HW/HEW after discharge	Yes	90	42.3
	No	123	57.7
Child vaccination status	Fully vaccinated	159	74.7
	Complete vaccinated	6	2.8
	Partially vaccinated	13	6.1
	Unvaccinated	35	16.4
Vitamin-A supplementation in the last 6 months	Yes	143	67.1
	No	70	32.9

HC, health center; HEW, health extension workers; HW, health workers; OTP, outpatient treatment program; SFP, supplementary feeding program.

### Child feeding practices and housing conditions of study participants

**FIGURE 2:**
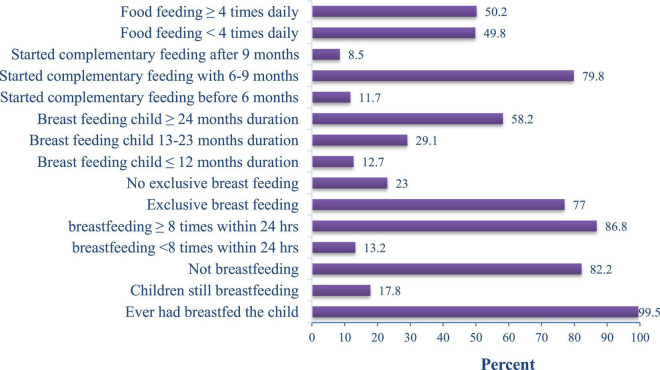
Child feeding practice of mothers/caregivers in Habro Woreda, Eastern Ethiopia, 2021 (*n* = 213).

Almost all (99.5%) mothers had ever breastfed their children, and 17.8% were still breastfeeding during the survey. Among currently breastfeeding mothers, 86.8% breastfed their children eight and more times in 24-h. About 77% of children were exclusively breastfed for 6 months, with an average duration of 5.8 (± 1.2 SD) months. More than three-quarters (79.8%) began complementary feeding between 6 and 9 months and 49.8% fed their children less than four times per day (Figure 2). In the present study, 65.3% of the households had latrines and 56.8% got drinking water from piped sources. Less than one-third (30.5%) of the households were getting their food from agricultural production, and there was no food supply for half (49.8%) at the time of the survey (Table 3).

**TABLE 3:** Housing conditions of house-holds of children discharged from SAM stabilization centers in Habro Woreda, Eastern Ethiopia, 2021 (*N* = 213).

Variables	Category	Frequency	Percent
Source of drinking water	Piped	121	56.8
	Surface water	41	19.3
	Unprotected spring	25	11.7
	Unprotected well	19	8.9
	Protected spring and well	7	3.3
Type of latrine exists	Private pit/wood slab	105	49.3
	Private pit/cement slab	30	14.1
	Shared VIP latrine	4	1.8
	Have no latrine	74	34.7
Source of food for the household	Agricultural production	65	30.5
	Purchase	122	57.3
	PSNP	18	8.5
	GFD	8	3.7
Household food stoke	No stock	106	49.8
	Enough up to 1 month	53	24.9
	Enough for 2–3 months	54	25.3

VIP, ventilator improved pit; GFD, general food distribution; PSNP, productive safety net program.

### Morbidity status and health seeking behavior of study participants

**FIGURE 3:**
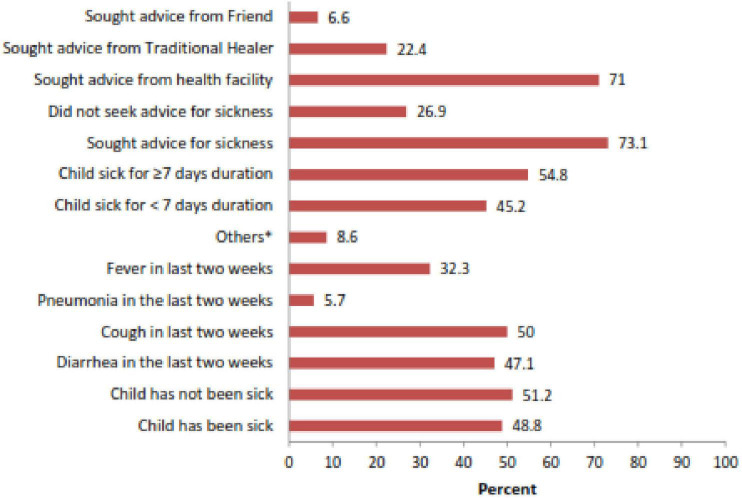
Morbidity status and Health-seeking behavior of the study participants in Habro Woreda, Eastern Ethiopia, 2021 (*n* = 213). *Others: burns, vomiting, ear infection, and scabies.

In the current study, 48.8% of children had been sick in the previous two weeks. The mean duration of the child illness was 8.8 (SD ± 8.4) days. Among the sick children, 47.1 and 50% had diarrhea and cough, respectively. Nearly three-fourths (73.1%) of mothers with sick babies sought advice, and 71% of them got advice from healthcare providers (Figure 3).

### Magnitude of relapse of acute malnutrition

**FIGURE 4:**
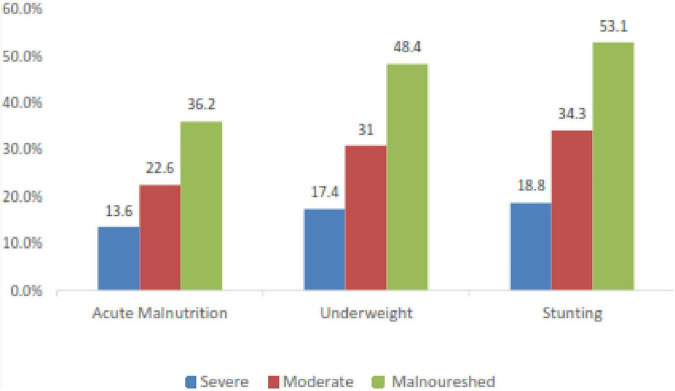
Nutritional status of children discharged from SAM stabilization centers in Habro Woreda, Eastern Ethiopia, 2021 (*n* = 213).

The overall magnitude of relapse of acute malnutrition among children following discharge from SC was found to be 36.2% [95% CI: 29.6,42.6]. Among acutely malnourished children, 13.6% were severely malnourished and 4.2% had bilateral pitting edema. In the current study, 53.1 and 48.4% of the children were stunted and underweighted, respectively (Figure 4).

### Factors associated with relapse of acute malnutrition

In the multivariate logistic regression analysis, MUAC at admission, latrine availability, follow-up visits after discharge, vitamin A supplementation in the past 6 months, household food security status, dietary diversity, and wealth index were significantly associated with relapse of acute malnutrition. In the current study, the odds of relapse of acute malnutrition among children admitted with MUAC < 110 mm was [AOR = 2.80; 95% CI: 1.05, 7.92] and children from households with no latrines were 2.5 times [AOR = 2.50; 95% CI: 1.08,5.65] more acutely malnourished than their counterparts. Children who were not visited by healthcare workers after discharge and not supplemented with vitamin A in the past 6 months were 2.81 times [AOR = 2.81; 95% CI: 1.15,7.22] and 3.4 times [AOR = 3.40; 95% CI: 1.40,8.09] more acutely malnourished than their counterparts, respectively. Similarly, children whose dietary diversity was less than four food groups were 3.1 times [AOR = 3.10; 95% CI: 1.31,7.33] more acutely malnourished than children who had greater than or equal to four food groups. In addition, children from food-insecure households were 4.51 times [AOR = 4.51; 95% CI: 1.40,15.06] more acutely malnourished than their counterparts. Moreover, the odds of relapse of acute malnutrition among the poorest wealth quintile was [AOR = 3.90; 95% CI: 1.23, 12.43] (Table 4).

**TABLE 4:** Factors associated with relapse of acute malnutrition among children discharged from stabilization centers in Habro Woreda, Eastern Ethiopia, 2021 (***N*** = 213).

Variables	Category	Acute malnutrition		COR (95% CI)	AOR (95% CI)
		**Yes = 77**	**No = 136**		
		***n* (%)**	***n* (%)**		
Total family size	1–3 members	4 (25.0)	12 (75.0)	1	1
	4–6 members	31 (28.7)	77 (71.3)	1.21 (0.36, 4.03)	1.81 (0.24,14.35)
	≥7 members	42 (47.2)	47 (52.8)	2.68 (0.80, 8.95)	3.20 (0.41, 24.99)
Maternal educational status	No formal education, unable to read and write	64 (43.8)	82 (56.2)	3.59 (1.29, 9.96)	1.0 (0.15, 6.00)
	No formal education, can read and write	8 (20.5)	31 (79.5)	1.19 (0.34, 4.10)	0.60 (0.08, 4.77)
	Primary school	5 (17.9)	23 (82.1)	1	1
Family wealth index	Poor	39 (54.9)	32 (45.1)	4.96 (2.34, 10.48)	3.90 (1.23,12.43)*
	Medium	24 (33.8)	47 (66.2)	2.08 (0.96, 4.46)	1.79 (0.60,5.42)
	Rich	14 (19.7)	57 (80.3)	1	1
Household water source	Non-improved	44 (51.8)	41 (48.2)	3.08 (1.72, 5.52)	1.41 (0.61, 3.36)
	Improved	33 (25.8)	95 (74.2)	1	1
Latrine availability	Yes	32 (23.0)	107 (77.0)	1	1
	No	45 (60.8)	29 (39.2)	5.18 (2.82, 9.56)	2.50 (1.08,5.65)*
Home visits by healthcare workers	Yes	17 (18.9)	73 (81.1)	1	1
	No	60 (48.8)	63 (51.2)	4.09 (2.16, 7.72)	2.81 (1.15, 7.22)*
Child immunization status	Yes	49 (30.8)	110 (69.2)	1	1
	No	28 (51.8)	26 (48.2)	2.42 (1.28,4.54)	1.91 (0.74, 5.25)
Vit-A supplementation in the past 6 months	Yes	35 (24.5)	108 (75.5)	1	1
	No	42 (60.0)	28 (40.0)	4.63 (2.51, 8.53)	3.40 (1.40, 8.09)*
Age at the start of complementary feeding	6–9 months	53 (31.2)	117 (68.8)	1	1
	≥9 months	11 (61.1)	7 (38.9)	3.47 (1.27, 9.44)	2.80 (0.59, 13.12)
	<6 months	13 (52.0)	12 (48.0)	2.39 (1.02, 5.59)	3.0 (0.85, 10.79)
Frequency of feeding in a day	<4 times	43 (40.6)	63 (59.4)	1.47 (0.83, 2.57)	2.41 (0.94, 6.43)
	≥4 times	34 (31.8)	73 (68.2)	1	1
Amount of feeding per day	<4 coffee cups	42 (49.4)	43 (50.6)	2.60 (1.45, 4.62)	1.40 (0.54, 3.56)
	≥4 coffee cups	35 (27.3)	93 (72.7)	1	1
Exclusive breastfeeding	Yes	54 (32.9)	110 (67.1)	1	1
	No	23 (46.9)	26 (53.1)	1.80 (0.94, 3.44)	1.90 (0.69, 5.71)
Household food security status	Food secure	8 (17.8)	37 (82.2)	1	1
	Food insecure	69 (41.1)	99 (58.9)	3.22 (1.41, 7.34)	4.51 (1.4, 15.06)*
MUAC at admission	<110 mm	33 (55.9)	26 (44.1)	4.94 (2.41, 10.15)	2.80 (1.05, 7.92)*
	110–115 mm	25 (41.0)	36 (59.0)	2.70 (1.32, 5.54)	2.76 (0.94, 7.75)
	>115 mm	19 (20.4)	74 (79.6)	1	1
Child dietary diversity	≥4 food groups	24 (20.8)	91 (79.1)	1	1
	<4 food groups	53 (54.1)	45 (45.9)	4.47 (2.45, 8.14)	3.10 (1.31, 7.33)*
History of diarrhea in the last 2 weeks	Yes	26 (53.1)	23 (46.9)	2.50 (1.3, 4.8)	2.0 (0.73, 5.62)
	No	51 (31.1)	113 (68.9)	1	1

*Significantly associated variables at *p*-value < 0.05.

CI, confidence interval; COR, crude odds ratio; AOR, adjusted odds ratio; MUAC, mid-upper arm circumference.

## Discussion

In the current study, children were assessed after an average of 10.9 months of discharge. Variables such as MUAC at admission, follow-up visits after discharge, dietary diversity, household food security status, vitamin A supplementation in the past 6 months, latrine availability and wealth index were identified as being associated with relapse of acute malnutrition.

One in three children was acutely malnourished after discharge from inpatient treatment programs in this study. This finding is consistent with studies conducted in the South Gondar zone, Ethiopia (27), and the Democratic Republic of Congo (41). However, is lower than the finding of study from Kenya (42). The discrepancy could be due to the small sample size in the current study, the difference in study design and length of time after discharge. In disparity, is higher than the findings from Burkina Faso (23), Nigeria (43), and Bangladesh (29). The reason for discrepancy could be due to the difference in sample size, nutrition intervention after discharge and socioeconomic characteristics. This suggests the requirement for appropriate nutrition education, counseling and follow-up of care after discharge to reduce the burden of relapse.

Children with MUAC < 110 mm at admission were more likely to relapse acute malnutrition than their counterparts. This finding is consistent with studies conducted in Burkina Faso (23), Nigeria (44), and Nepal (45). The possible reason for this consistency could be, children admitted for SAM treatment and discharged as recovered might have chances to develop a new episode of acute malnutrition. Continued nutritional support, potentially in the form of a supplementary feeding program, may contribute to lower acute malnutrition rates (46).

In the current study, the absence of latrine was associated with relapse of acute malnutrition. Children from households with no latrine were more acutely malnourished than their counterparts. This finding is supported by the study conducted in the Afar region, Ethiopia (4). This could be, due to the fact that good sanitation could prevent contamination of the environment by excreta and the transmission of pathogens from the feces of an infected person to a new host (47). In contrast, poor environmental sanitation is an important cause of infectious diseases, especially diarrhea and intestinal parasite; which contributes to malnutrition (48).

According to the WHO recommended schedules, children discharged from inpatient programs should be followed by healthcare workers at 1, 3 and 6 months, and then twice yearly until the child is at least three years old (1). However, such a follow-up was not routinely done in the current study. Children who were not visited by healthcare workers after discharge were more acutely malnourished than their counterparts. This finding is supported by the study conducted in the South Gondar zone, Ethiopia (27). The result indicates that follow-up visits after discharges are very crucial for the successful management of acute malnutrition, since some cases relapsed or developed comorbidities during this period. This might be due to lack of organized or insufficient capacity of health system to follow-up after discharges. Even though there are health extension workers in this setting, there might be lack of coordination and communication gap between health extension workers and health facilities providing inpatient treatment of SAM.

In the current study, children who did not receive vitamin-A supplementation in the past 6 months were more acutely malnourished than their counterparts. This finding is similar to the study conducted in the South Gondar zone, Ethiopia (27). This could be, due to the protective role of vitamin A in promoting and regulating activities in both the inborn and adaptive immune system, thus enhancing immune function, is a rationale for supplementing every 6 months (49, 50).

Children whose dietary diversity was below four food groups were more acutely malnourished compared to children with greater than or equal to four diversified foods. This finding is consistent with studies conducted in Pakistan and Bangladesh (19, 51). The reason for this consistency could be that a household’s capability to acquire necessary foods and general availability of food is a precondition to achieving the diversification of children’s diets (52). Overall, children’s food in this study seemed monotonous, primarily cereal-based. Additionally, since children are in a period of rapid growth and development, failure to supply adequate and diversified food for daily bodily demands would result in growth faltering. In the current study, relapse of acute malnutrition after discharge was related to household (HH) food insecurity status. The odds of relapse among children in food insecure HHs was higher than in food secure HHs. This finding is in line with studies conducted in the Jimma zone, Ethiopia (14), South Gondar zone, Ethiopia (27), and Nigeria (53). Food insecurity can be linked to insufficient intake of diversified foods and studies have reported consumption of less diversified food as being associated with acute malnutrition (14). In addition, children who have a practice of feeding less diversified diet are easily exposed to infection due to poor immunity.

The low socioeconomic status of the HH increased the likelihood of being more acutely malnourished. In the present study, children from HHs with poor wealth index were more acutely malnourished than children from HHs with high wealth index. This finding agreed with studies conducted in Haramaya district, Ethiopia (15), Hawassa, Ethiopia (28), and India (54). This could be justified by, the low-income levels of the HHs limiting the type and the amounts of food available for consumption which in turn may lead to feed children less diversified diets.

Despite these important merits, the study had some limitations. Primarily, due to the nature of the cross-sectional study design, it was difficult to establish the cause-effect relationship between the study variables. Secondly, the small sample size used in the study may not represent a larger population. In addition, recall bias was one of the limitations of the study since some questions were asked about the events that occured 24-h back. This was minimized by probing the respondents about the events.

## Conclusion

The magnitude of relapse of acute malnutrition following discharge from inpatient treatment programs was very high. One in three children was acutely malnourished after discharge in Habro Woreda, Eastern Ethiopia, alarming for the requirements of appropriate nutrition education, counseling and follow-up of care. MUAC at admission, follow-up visits after discharge, dietary diversity, household food security status, vitamin A supplementation in the past 6 months, latrine availability, and wealth index were predictors of relapse of acute malnutrition. Therefore, programmers and stakeholders working on nutrition should design interventions that focus on improving household food insecurity through public Safety Net programs should be strengthened, and nutrition counseling and education, are crucial to protect children against post-discharge relapse of acute malnutrition. Moreover, healthcare workers should give due emphasis to organized continuous follow-up and periodic monitoring for early detection of those at risk of relapse, especially during the first 6 months of discharge, to reduce and control the risk of relapse of acute malnutrition. The relapse rate should be a performance indicator of stabilization centers. Furthermore, a study that focuses on operational and behavioral change is recommended to fill the gap in the study area.

## Data availability statement

The original contributions presented in this study are included in the article/supplementary material, further inquiries can be directed to the corresponding author.

## Ethics statement

The study was conducted following the principles of the Helsinki Declaration. The studies involving human participants were reviewed and approved by Haramaya University, College of Health and Medical Sciences Institutional Health Research Ethics Review Committee (IHRERC). Written informed consent to participate in this study was provided by the participants or their legal guardian/caretakers of the children.

## Author contributions

MA, BHA, and KTR conceived, designed the study, acquired the data, analyzed, and interpreted the findings. IK, SH, ATG, YB, and AM revised and provided the critical intellectual feedback. AM and IK drafted the manuscript. All authors have read and approved the final manuscript for submission.
